# Stable Inheritance of Transgene and Yellow Fluorescent Protein Gene Expression in Progeny of Transgenic Cacao (*Theobroma cacao*) Plants

**DOI:** 10.3390/plants15040642

**Published:** 2026-02-18

**Authors:** George Austin, Jesse Jones, Abigail Stevens, Elaine Zhang, Taylor Thompson, Michael Gomez, Geoffrey Vrla, Youngbin Oh, Jean-Philippe Marelli, Carl M. Jones, Brian Staskawicz, Myeong-Je Cho

**Affiliations:** 1 Innovative Genomics Institute, University of California, Berkeley, CA 94720, USA; austing@berkeley.edu (G.A.); jessemjones@gmail.com (J.J.); abbey.stevens@berkeley.edu (A.S.); ezhang902@gmail.com (E.Z.); taycthompsonn@gmail.com (T.T.); michaelgzrs@gmail.com (M.G.); gvrla@berkeley.edu (G.V.); oybin@berkeley.edu (Y.O.); stask@berkeley.edu (B.S.); 2 Mars Incorporated, Davis, CA 95616, USA; jean-philippe.marelli@effem.com (J.-P.M.); carl.jones@effem.com (C.M.J.); 3 Department of Plant and Microbial Biology, University of California, Berkeley, CA 94720, USA

**Keywords:** *Theobroma cacao*, *Agrobacterium tumefaciens*, genetic transformation, somatic embryogenesis, transgene expression and inheritance, plant phenotype

## Abstract

Genetic engineering tools have the potential to rapidly and precisely improve the genome of slow-to-breed cacao. We previously developed an efficient protocol for transforming cacao using cotyledonary explants derived from secondary somatic embryos via *Agrobacterium tumefaciens*. In this study, we demonstrate that our transformation protocol is successful in elite cultivars, INIAPG-038 and Matina 1-6, producing fertile seeds with stable visual marker inheritance regardless of whether the transgenic plants were used as the pollen or ovule donor. Three vectors were used in the transformations, each containing genes for *enhanced yellow fluorescent protein* (*eyfp*) and *neomycin phosphotransferase* II (*npt*II). Three transgenic INIAPG-038 events and one transgenic Matina 1-6 event were used to evaluate seed fertility and the stability of transgene inheritance in cacao seeds and plants. The T_1_ progeny of these four transgenic events were analyzed for YFP expression and transgene presence. YFP expression segregated at a 1:1 ratio in all events when the transgenic plants were crossed with non-transgenic plants, while a 3:1 segregation was observed when transgenic events were crossed with each other. The transgenic plants exhibited a normal phenotype compared to non-transgenic control plants, producing seeds with a 97% germination rate.

## 1. Introduction

The global multi-billion-dollar chocolate industry is based on the fruit of cacao (*Theobroma cacao* L.) trees. Cacao is predominantly cultivated in West Africa, followed by Southeast Asia and South America, where it was originally domesticated [1]. There are ten recognized genetic groups of cacao [2]. The most flavorful groups, Criollo and Nacional, suffer from susceptibility to disease and pests [3] as well as an accumulation of yield-impacting deleterious mutations [4]. Despite a price premium for Criollo varieties with floral, fruity notes, highly productive Amelonado hybrids dominate the global market. Globally, cacao production is greatly reduced by diseases, whether affecting Criollo and Nacional in South America or Amelonado in West Africa [5]. Developing new cacao cultivars that combine high yield, quality, and resistance to diseases and pests among different genetic groups using traditional breeding methods is a time-consuming and complex process.

As is the case with most fruit tree species, traditional plant breeding of cacao can take 20 or more years to release a new cultivar [6] and is hampered by lengthy juvenile periods, high heterozygosity, self-incompatibility, and a lack of funding [7,8]. On the other hand, genetic modification using *Agrobacterium* and biolistic methods, which allows for the addition of valuable genes not naturally present within a species, has achieved dramatic crop improvements such as resistance to disease, pests, abiotic stress, and herbicides, along with enhanced nutrient use efficiency and improved photosynthesis, ultimately resulting in increased yields [9]. This approach overcomes the challenges associated with traditional breeding. In addition, recent genome editing technology enables the precise modification of genes or regulatory sequences already present within a species, providing an expanded toolkit for trait development and genetic discovery. These genome modifications can alter expression levels, facilitate the discovery of novel alleles, manipulate quantitative trait loci [10], and generate knockout lines for studying gene function. The first stable transformation of cacao occurred in the PSU SCA-6 cultivar, resulting in the production of six pods containing 143 transgene-positive and 139 transgene-negative seeds from a single transgenic event [11]. In a previous study [12], we reported the successful transformation of INIAPG-038, a high-yielding elite breeding line developed by Mars Wrigley, Incorporated, in collaboration with the United States Department of Agriculture (USDA) and Instituto Nacional de Investigaciones Agropecuarias (INIAP). This line also has some resistance to the fungal diseases *Moniliophthora roreri* (frosty pod) [13] and *Moniliophthora perniciosa* (witches’ broom) [14], which are widespread in Central and South America [15]. Matina 1-6, which has been sequenced, belongs to the most widely planted Amelonado genetic group [16]. It is susceptible to cacao swollen shoot virus (CSSV) [17], which is prevalent in West Africa, where it is commonly cultivated. There are no previous reports in the literature of successful transformation in Matina 1-6.

Although genetic transformation can be a powerful tool, unintended detrimental changes may take place during the tissue culture and transformation process. Mutations, including those causing sterility, can arise due to somaclonal variation, where stress during tissue culture can cause DNA methylation, activation of transposable elements, or the creation of new mutations [18,19]. The expression of the inserted genes may be affected by nearby endogenous genes, complex transgene loci, and excessive expression [20]. Inserted genes may interfere with important endogenous genes that are located nearby, bisected by, or homologous to the transgene [21,22,23]. Transgene silencing can also be an issue, occurring at the transcriptional level through DNA methylation or post-transcriptionally through RNA degradation [24]. Due to these potential issues, as well as chimerism and multiple copy insertions, the transgene inheritance ratio expected when the T_0_ is outcrossed may skew away from the expected Mendelian segregation ratio. It is crucial to screen novel transgenic lines for all these issues through T_0_ and T_1_ plant analysis to ensure that the genetic transformation was successful. In cacao, germplasm is commonly disseminated through polyclonal seed gardens, producing hybrid seeds in West Africa, and through clonal cuttings in the Americas and Asia [25]. In both instances, the stable expression and inheritance of transgenes are important for creating new genetically engineered cultivars or breeding lines. Once stably integrated, transgene inheritance and the stability of mRNA and protein expression levels will remain constant over generations, even when backcrossed or outcrossed [26].

In this study, we demonstrate that our genetic transformation protocol for two cacao cultivars, INIAPG-038 and Matina 1-6, is successful in producing plants with stably incorporated transgenes and stable transgene expression. Importantly, the transformation process did not adversely affect seed fertility, pollen and ovule viability, or plant phenotype. These findings were confirmed through evaluation of plant morphology, cross-pollination compatibility, seed size, germination rate, and transgene expression and inheritance in both T_0_ and T_1_ plants.

## 2. Results and Discussion

### 2.1. Generation of Transgenic Cacao Plants

**Figure 1:**
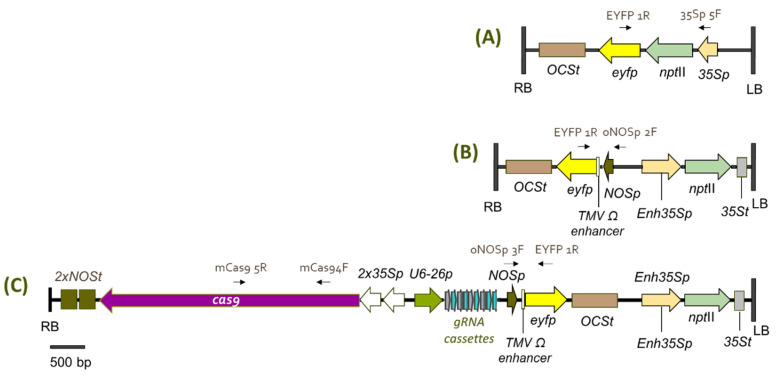
Schematic diagram of three transformation vectors used for *Theobroma cacao* L. transformation. (**A**) pDDNPTYFP-1 contains a 3637 bp T-DNA fragment with a translational fusion of the neomycin phosphotransferase II (*npt*II) and enhanced yellow fluorescent protein (*eyfp*) gene, driven by a CaMV 35S promoter. (**B**) pDDNPTYFP-2 contains a 4416 bp T-DNA fragment with two gene cassettes: *eyfp* is driven by a Nos promoter/TMV Ω enhancer, and *npt*II is driven by an enhanced CaMV 35S promoter. (**C**) pMGCC3YFP contains an 11,460 bp T-DNA fragment with four gene cassettes: *eyfp* is driven by a Nos promoter/TMV Ω enhancer, *npt*II is driven by an enhanced CaMV 35S promoter, and two additional cassettes encode *cas9* and *gRNA*.

The primary goal of this research is to confirm whether transgenic cacao plants generated using our protocol [12] are healthy, phenotypically normal, fertile, and capable of transmitting inserted transgenes in a Mendelian fashion without any adverse effects. To evaluate stable transgene expression and inheritance, three independent transgenic events of INIAPG-038 and one transgenic event of Matina 1-6 were analyzed. One event (INIAPG-038 EVT1) was transformed with the pDDNPTYFP-1 vector (Figure 1A), while two events (INIAPG-038 EVT2 and EVT3) were transformed with the pDDNPTYFP-2 vector (Figure 1B). All three were generated previously via *Agrobacterium*-mediated transformation [12]. In contrast, Matina 1-6 EVT1 was generated for this study using the pMGCC3YFP vector, which contains *npt*II and *eyfp* gene cassettes (Figure 1C), following the same transformation protocol [12]. It also contains *SpCas9* and gRNA cassettes targeting disease resistance, which is not evaluated in this study.

The transformation frequency in INIAPG-038 was 3.7% at the T_0_ plant level (three independent events from 82 secondary somatic embryos) [12], whereas Matina 1-6 yielded one event from 162 embryos (0.62%). The low transformation frequency observed in Matina 1-6 may be attributable to reduced T-DNA delivery efficiency [12] and the larger T-DNA region used in this study (Figure 1). Because these results are based on a limited number of events, larger-scale transformation experiments using the same construct would be necessary to more definitively assess potential differences in transformation frequency between these two cultivars.

### 2.2. Molecular Confirmation of Transgene Integration

**Figure 2:**
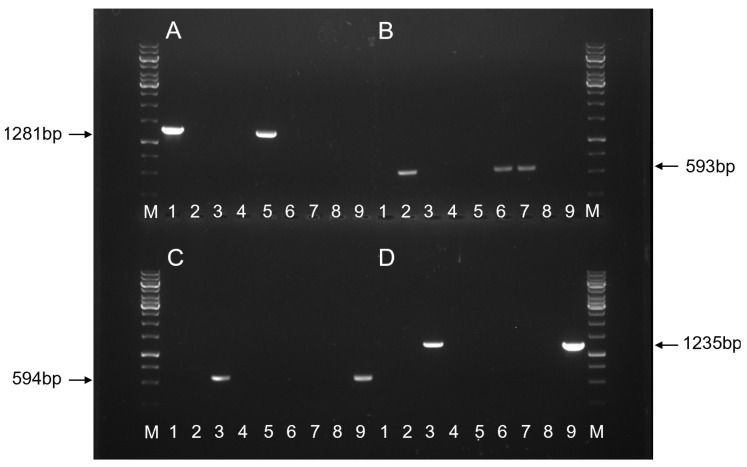
PCR analysis of genomic DNA from four different transgenic *Theobroma cacao* L. events. Genomic DNA was extracted from the leaf tissues of one non-transgenic INIAPG-038 control, three transgenic INIAPG-038 events (EVT1, EVT2, and EVT3), one non-transgenic Matina 1-6 control, and one transgenic Matina 1-6 event (EVT1). M, GeneRuler 1 kb DNA ladder; Lane 1, pDDNPTYFP-1 plasmid; Lane 2, pDDNPTYFP-2 plasmid; Lane 3, pMGCC3YFP plasmid; Lane 4, non-transgenic INIAPG-038; Lane 5, INIAPG-038 EVT1; Lane 6, INIAPG-038 EVT2; Lane 7, INIAPG-038 EVT3; Lane 8, non-transgenic Matina 1-6; Lane 9, Matina 1-6 EVT1. (**A**) Amplified 1281 bp fragment products using 35Sp 5F and EYFP 1R. (**B**) Amplified 593 bp fragment products using oNOSp 2F and EYFP 1R. (**C**) Amplified 594 bp fragment products using oNOSp 3F and EYFP 1R. (**D**) Amplified 1235 bp fragment products using mCas9 4F and mCas9 5R.

Unique primer sets were designed for each construct (Table S1) and validated through polymerase chain reaction (PCR) analysis using plasmid DNA and genomic DNA extracted from the leaf tissues of non-transgenic plants and transgenic events (Figure 2). The presence of the eyfp transgene was confirmed by PCR using primer sets, 35Sp 5F/EYFP 1R, oNOSp 2F/EYFP 1R, and oNOSp 3F/EYFP 1R for pDDNPTYFP-1, pDDNPTYFP-2, and pMGCC3YFP vectors, respectively (Figure 1), as well as their corresponding transgenic events, INIAPG-038 EVT1 (Figure 2A), INIAPG-038 EVT2/EVT3 (Figure 2B), and Matina 1-6 EVT1 (Figure 2C). For Matina 1-6 EVT1, the cas9 transgene was also confirmed using the mCas9 4F/mCas9 5R primer set (Figure 2D). A 1281 bp amplified product corresponding to the CaMV 35S promoter::nptII::eyfp gene fragment was detected in the genomic DNA of INIAPG-038 EVT1 using the 35Sp 5F/EYFP 1R primer set (Figure 2A; Table S1), while a 593 bp NOS promoter::TMV Ω enhancer::eyfp gene fragment was amplified from INIAPG-038 EVT2 and INIAPG-038 EVT3 using the oNOSp 2F/EYFP 1R primer set (Figure 2B; Table S1). In Matina 1-6 EVT1, a 594 bp NOS promoter::TMV Ω enhancer::eyfp gene fragment was amplified using the oNOSp 3F/EYFP 1R primer set (Figure 2C; Table S1), and/or a 1235 bp cas9 fragment was amplified using the mCas9 4F/mCas9 5R primer set (Figure 2D; Table S1).

### 2.3. YFP Expression in T_0_ Plants

All four T_0_ events exhibited YFP expression in both leaf and root tissues (Figure S1). However, visual YFP expression driven by the CaMV35S promoter in INIAPG-038 EVT1 was notably stronger than that of the other three events—INIAPG-038 EVT2, EVT3, and Matina 1-6 EVT1—where expression was driven by the NOS promoter with the TMV Ω enhancer (Figure S1). Western blot analysis showed that YFP protein levels driven by the CaMV35S promoter were also 10- to 30-fold higher than those driven by the NOS promoter with the TMV Ω enhancer (Figure S2). This is similar to findings by Sanders et al. [27], who reported that the CaMV35S promoter facilitated a 30-fold higher expression of the *npt*II gene in petunias compared to the NOS promoter. Although the TMV Ω enhancer has also been shown to increase transgene expression in plants by 3% when attached to the CaMV35S promoter [28] and by 100% when attached to the heat shock protein (*hsp80*) promoter [29], in our study, the NOS promoter with the TMV Ω enhancer did not surpass the YFP expression levels of the CaMV35S promoter (Figure S1). In INIAPG-038 EVT2 and EVT3, YFP expression highlights the leaf veins, making them clearly visible, whereas in Matina 1-6 EVT1, the YFP expression is more evenly distributed between veins and interveinal areas (Figure S1). Since all three events share the same NOS promoter::TMV Ω enhancer, the observed differences in YFP expression are likely attributable to variations in integration site, copy number, or structural modifications to the promoter or terminator regions.

### 2.4. Fertility and Seed Set in T_0_ Plants

**Figure 3:**
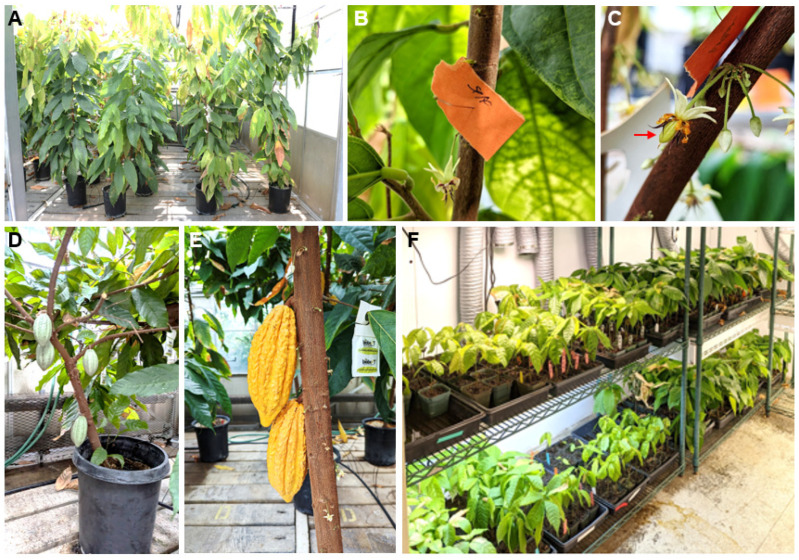
T_0_ and T_1_ cacao plants, flowers, fruits, and germination test used in this study. (**A**) Non-transgenic plants (left two rows) and T_0_ INIAPG-038 plants (right two rows). (**B**) Flowers three days after self-pollination on a non-transgenic INIAPG-038. (**C**) A fruit six days after pollination on a non-transgenic Matina 1-6 ovule pollinated with non-transgenic INIAPG-038 pollen. (**D**) Fruits from self-pollinated Matina 1-6 EVT1 approximately three months after pollination. (**E**) Fruits resulting from a cross between INIAPG-038 EVT1 and Matina 1-6 EVT1. (**F**) Germination test using T_1_ seeds from crossed fruits.

To evaluate seed fertility and transgene expression and inheritance in progeny plants, transgenic cacao flowers were hand-pollinated with transgenic or non-transgenic counterparts using plants approximately 3 to 4 years post-transformation. (Figure 3A,B). Fruit development was monitored, and resultant seeds were planted (Figure 3C–F). In nature, insects play a key role in cacao pollination, particularly for self-incompatible plants [30]. Self-pollination of INIAPG-038 did not produce any pods due to self-incompatibility, while self-pollination of Matina 1-6 yielded pods, regardless of whether the plants were transgenic or non-transgenic (Table 1). Two genetic regions have been identified for causing self-incompatibility in cacao. One blocks fertilization, and the other causes gametic non-fusion and early fruit drop [31]. Matina 1-6 is from the Amelonado genetic group, which is known for having alleles conferring self-compatibility [16], while INIAPG-038 has a mixed heritage, including the Nacional group, which is known to have a low incidence of self-compatibility alleles [31]. In this study, self-compatibility in non-transgenic Matina 1-6 and self-incompatibility in non-transgenic INIAPG-038 were unaffected by transformation (Table 1).

### 2.5. YFP Expression and Inheritance in T_1_ Progeny

**Figure 4:**
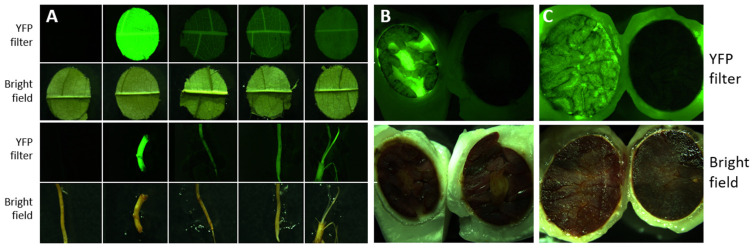
YFP expression in progeny tissues derived from crosses between non-transgenic and T_0_ *Theobroma cacao* L. plants. (**A**) Top row and third row: YFP filter; second row and bottom row: brightfield. From left to right: progeny leaf and root samples from crosses of non-transgenic INIAPG-038, INIAPG-038 EVT 1, INIAPG-038 EVT 2, INIAPG-038 EVT 3, and Matina 1-6 EVT1, each crossed with non-transgenic Matina 1-6. (**B**) Seeds from a cross between a non-transgenic Matina 1-6 ovule donor and a transgenic INIAPG-038 EVT1 pollen donor, showing a YFP-positive (transgenic; left) and a YFP-negative (non-transgenic; right) seed. (**C**) Seeds from a cross between a transgenic INIAPG-038 EVT1 ovule donor and a non-transgenic Matina 1-6 pollen donor, showing a YFP-positive (transgenic; left) and a YFP-negative (non-transgenic; right) seed. The pulp surrounding the seeds seen in (**C**) expresses YFP expression, consistent with its maternal origin from the transgenic ovule donor.

Differences in the expression of YFP among T_1_ seeds and plants were observed across the transformation vectors, as expected. The transgenic progeny of INIAPG-038 EVT1, which utilized the CaMV 35S promoter for YFP, exhibited very strong YFP expression in all tissue types. In the leaf and root tissues of T_1_ plants from crosses of non-transgenic INIAPG-038, INIAPG-038 EVT1, INIAPG-038 EVT2, INIAPG-038 EVT3, and Matina 1-6 EVT1 with non-transgenic Matina 1-6 (Figure 4A), the level and pattern of YFP expression were the same as those of their individual T_0_ counterparts (Figure S1). INIAPG-038 EVT1 transgenic progeny, carrying the CaMV 35S promoter for YFP, exhibited significantly stronger YFP expression compared to the T_1_ progeny of INIAPG-038 EVT2, EVT3, and Matina 1-6 EVT1, which utilize the NOS promoter/TMV Ω enhancer for YFP (Figure 4A). In the seed of INIAPG-038 EVT1, YFP expression was visible after manually removing the fruit pulp and seed coat or more rapidly by slicing the seed in half (Figure 4B). In contrast, seeds from crosses with INIAPG-038 EVT2, EVT3, and Matina 1-6 EVT1, all of which had the (NOS) promoter::TMV Ω enhancer for YFP, did not show visible YFP expression in the kernel but exhibited expression in the radicle and first true leaves after seed germination. Strong YFP expression was observed in a YFP-positive seed resulting from a cross with a non-transgenic Matina 1-6 ovule donor (Figure 4B). Seeds from a cross between a transgenic INIAPG-038 EVT1 ovule donor and a non-transgenic Matina 1-6 pollen donor (Figure 4C) also exhibit YFP expression, but the YFP expression in the pulp surrounding the seeds is determined by the source of the ovule donor plant (Figure 4B,C).

**Figure 5:**
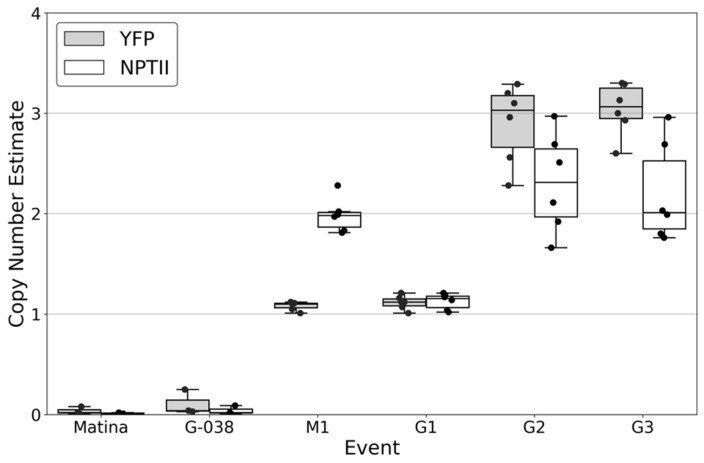
Copy number estimate of YFP and NPTII in T_0_ *Theobroma cacao* L. plants. Calculations of YFP and NPTII copy number were performed using the Bio-Rad QX200 digital droplet PCR (ddPCR) system and QuantaSoft Analysis software (quantasoft version 1.7). Copy number estimate refers to the target counts of the transgene divided by 0.5× target counts of the TcEF1a reference gene. Values are the mean and interquartile range of three separate measurements of two leaf samples. Matina, non-transgenic Matina 1-6; G-038, non-transgenic INIAPG-038; M1, transgenic Matina 1-6 EVT1; G1, transgenic INIAPG-038 EVT1; G2, transgenic INIAPG-038 EVT2; G3, transgenic INIAPG-038 EVT3.

YFP expression in T_1_ progeny tissues followed expected Mendelian segregation ratios for all crosses (Table 1). PCR analysis of progeny from selected crosses was consistent with observations from visual inspections (Table S2). Crosses between transgenic and non-transgenic cacao plants from all four events demonstrated a 1:1 segregation ratio of YFP expression (Table 1), consistent with previous findings by Maximova et al. [11]. Copy-number analysis using ddPCR indicated that Matina 1-6 EVT1 and INIAPG-038 EVT1 each contain a single copy of *eyfp*, whereas INIAPG-038 EVT2 and EVT3 contain three copies (Figure 5), confirming that all four events in our study have single transgene integration sites. Detailed T-DNA insertion site characterization would further strengthen the molecular analysis and will be considered in future studies. Self-pollination of Matina 1-6 EVT1 yielded a 119:42 segregation, corresponding to the expected 3:1 ratio of YFP-expressing to non-YFP-expressing T_1_ progeny plants (Table 1). Visual YFP expression levels were low and similar between T_1_ progeny fixed (biallelic) for the transgene and those still segregating for it (Table 1 and Table S2).

Reciprocal crosses between INIAPG-038 EVT1 and Matina 1-6 EVT1 produced a segregation of 55:36:40 (strong/weak/no expression). Although this pattern did not follow an expected 1:1:1:1 ratio (strong/moderately strong/weak/no expression), no progeny plants with moderately strong expression were observed, and it instead reflected a 2:1:1 (strong/weak/no expression) ratio for expression level because the strong YFP expression driven by the CaMV 35S promoter in INIAPG-038 EVT1 overshadowed the contribution of the weaker NOS promoter::TMV Ω enhancer. As a result, progeny inheriting both transgenes displayed expression levels indistinguishable from those inheriting only the CaMV 35S-driven YFP (Table 1 and Table S2). Nevertheless, the overall positive-to-negative YFP segregation still aligned with the expected 3:1 ratio (Table 1 and Table S2). Crosses between INIAPG-038 EVT2 or EVT3 and Matina 1-6 EVT1, all expressing YFP under the NOS promoter::TMV Ω enhancer, demonstrated segregation ratios of 14:4 and 6:2, respectively, both consistent with a 3:1 pattern and characterized by uniformly weak expression (Table 1 and Table S2). In these crosses, visual YFP expression was comparable between progeny inheriting a single NOSp::Ω-driven transgene and those inheriting NOSp::Ω-driven transgenes from both parental events (Table S2), reflecting the low YFP expression levels of the parental events (Figure S1).

PCR analysis of the INIAPG-038 EVT1 × Matina 1-6 EVT1 and INIAPG-038 EVT2 × Matina 1-6 EVT1 crosses showed a 1:1:1:1 segregation ratio (Table S2). For the INIAPG-038 EVT1 × Matina 1-6 EVT1 cross, five plants amplified CaMV 35S promoter::*nptII::eyfp*, NOS promoter/TMV Ω enhancer/*eyfp* (oNOSp 3F/EYFP 1R), and cas9 fragments, ten amplified only the CaMV 35S promoter::*nptII::eyfp*, eight amplified the NOS promoter/TMV Ω enhancer/*eyfp* and *cas9*, and eight showed no amplification. Because the plasmid pMGCC3YFP, which was used to generate Matina 1-6 EVT1, carries the NOSp::Ω::eyfp–cas9 cassette (Figure 1C), plants with only these fragments displayed weak YFP expression (Table S2). Visual determination of these tested plants revealed fifteen plants with strong YFP expression driven by the CaMV 35S promoter from INIAPG-038 EVT1 with or without Matina 1-6 EVT1, eight with weak expression driven by the NOS promoter/TMV Ω enhancer from Matina 1-6 EVT1, and eight with no expression (Table S2). For the INIAPG-038 EVT2 x Matina 1-6 EVT1 cross, five plants showed amplification of both NOS promoter/TMV Ω enhancer/*eyfp* fragments using either (oNOSp 2F/EYFP 1R and oNOSp 3F/EYFP 1R) or *cas9*; four amplified the oNOSp 2F/EYFP 1R fragment only; five amplified the oNOSp 3F/EYFP 1R fragment and cas9; and four showed no amplification (Table S2). Visual results for these tested plants indicated that fourteen YFP-positive plants contain NOS promoter/TMV Ω enhancer/*eyfp* and/or *cas9*, along with four YFP-negative plants. In self-pollinated Matina 1-6 EVT1, PCR analysis revealed eighteen transgene-positive progeny and four negative progeny, corresponding to a 3:1 segregation ratio. These results are consistent with Mendelian inheritance of a single transgene locus.

### 2.6. Fertility, Seed Set, and Germination

Genetic transformation in plants can lead to unintended effects, including male sterility or low seed set, due to somaclonal variation and disruptions caused by the transformation process. The tissue culture and transformation process may introduce genetic and epigenetic changes that affect fertility [32,33], while transgene insertion or mutagenesis can disrupt endogenous genes critical for reproduction, leading to sterility or developmental abnormalities [34]. Male sterility can also result from disruptions to genes regulating pollen development. Additionally, overexpression of stress-related genes or disruption of hormonal pathways, such as auxins, gibberellins, or abscisic acids, can impair reproductive processes and seed development, resulting in low seed set [35,36]. However, in our study, all four transgenic cacao events set fruits and seeds without affecting seed set percentages, regardless of the transgenic nature of the pollen or ovule donor (Table 1). Additionally, the T_1_ seed sizes of transgenic and non-transgenic plants were not significantly different (Table 2).

Seeds produced by the crosses in this study germinated and formed phenotypically normal plants at a rate of 97% (Figure 3F, Table 3). Most pods harvested in this study contained a couple of seeds noticeably smaller than average; these also formed normal plants but took longer to do so. There were also a negligible number of undeveloped seed structures found within harvested pods that were not included in our results. As the entire cacao industry is based on the cacao seed yield, it is of utmost importance that seeds are not negatively affected as a result of the transformation process. Seed size appears to be unaffected by the transgenic nature of either INIAPG-038 or Matina 1-6 compared to non-transgenic null-segregant seed (Table 3). There also appears to be no correlation between transgene inheritance and germination rates or seed size within pods (Table 3), similar to results in transgenic pear seeds [37]. It is unlikely that seeds inheriting the transgene would be smaller, as that would require the genomic imprinting to be affected by the insertion of the transgene or the tissue culture process [38].

### 2.7. Conclusions

In conclusion, transgenic cacao plants generated from the elite cultivars INIAPG-038 and Matina 1-6 using our transformation protocol exhibited no detectable adverse phenotypic differences relative to their non-transgenic counterparts. Furthermore, segregation ratios for YFP expression confirmed the stable inheritance of the inserted transgenes. This transformation protocol thus provides a reliable approach for cacao improvement through genetic modification and gene editing techniques.

## 3. Materials and Methods

### 3.1. Plant Materials

Transgenic and non-transgenic cacao plants of INIAPG-038 and Matina 1-6 used in this study were generated using tissue culture and genetic transformation, as previously described by Jones et al. [12]. The donor material for these cultivars was shipped from the USDA ARS Subtropical Horticulture Research Station in Miami, FL. Three independent transgenic INIAPG-038 events were produced in a previous study; 1 event (INIAPG-038 EVT1) was transformed with pDDNPTYFP-1 (Figure 1A), and 2 events (INIAPG-038 EVT2 and INIAPG-038 EVT3) with pDDNPTYFP-2 (Figure 1B). The transgenic Matina 1-6 event (Matina EVT1) was generated using the pMGCC3YFP vector (Figure 1C) according to the protocol published by Jones et al. [12].

In total, for each non-transgenic plant, there were 2–3 trees. For transgenic Matina 1-6 EVT 1 and INIAPG-038 EVT 1, 2, and 3, there were 12, 11, 3, and 3 trees, respectively. At the time of pollination for this study, transgenic INIAPG-038 trees were approximately 4 years post-transformation and approximately 3 years since embryo germination, and transgenic Matina 1-6 trees were one year younger. Non-transgenic cacao trees were ~4 years old at the time of pollination.

### 3.2. Transformation Vectors

Three binary vectors, pDDNPTYFP-1, pDDNPTYFP-2, and pMGCC3YFP, each containing *eyfp* and *npt*II, were used for cacao transformation. pDDNPTYFP-1 contains a neomycin phosphotransferase II (*npt*II)-GlyLink-enhanced yellow fluorescent protein (*eyfp*) translational fusion, driven by the cauliflower mosaic virus 35S (CaMV 35S) promoter (35Sp) (Figure 1) [12], while pDDNPTYFP-2 contains 2 separate gene cassettes, *npt*II driven by the enhanced 35Sp and *eyfp* driven by the Nos promoter (NOSp)/TMV Ω enhancer (Figure 1B) [12]. Both vectors were built in the pCAMBIA2300 backbone. For pMGCC3YFP (Figure 1C), the pCAMBIA2300 binary vector backbone was assembled with a CRISPR expression system, as described in Gomez et al. [39]. Subsequently, the YFP expression cassette, consisting of a NOSp/NOSp/TMV Ω enhancer, *eyfp*, and OCS terminator, was PCR-amplified using Phusion HF Polymerase [New England Biolabs (NEB), Ipswich, MA, USA] and cloned into the *Kpn*I site of the binary vector using Gibson Assembly (NEB, Ipswich, MA, USA).

### 3.3. Plant Care

Plantlets were transferred from tissue culture into 10 cm pots filled with Sun Gro^TM^ potting mix (Sun Gro Horticulture Distribution Inc., Agawam, MA, USA) when they had developed 8–12 leaves. Plants were maintained in a growth chamber at 28 °C, 80% RH, 16 h photoperiod at 100–250 μmol m^−2^ s^−1^ produced by fluorescent bulbs for 1 month before being transferred to the greenhouse. In the greenhouse, plants were grown under white 40% netting shade cloth and repotted as needed, with final transplantation to 10-gallon pots. Greenhouse conditions were maintained at a minimum temperature of 20 °C without supplemental lighting. The plants were pruned to maintain a single trunk architecture, kept at a height of ~2.2 m, and spaced at 1.6 m^2^ per plant. Plants were automatically fertigated twice a day to keep the potting mix constantly moist. Fertigation water contained a mix of Peter’s 20-20-20 (ICL Specialty Fertilizers, Summerville, SC, USA) and calcium nitrate (Yara, Tampa, FL, USA) at 1.5 E.C., supplemented monthly with 50 g of the slow-release fertilizer Osmocote plus (ICL Specialty Fertilizers, Summerville, SC, USA). Soil electrical conductivity (E.C.) was monitored, and additional fertilizer was aqueously applied when E.C. fell below 1.2 dS m^−1^.

### 3.4. Pollination

Around thirty-six months post-transformation and eighteen months after soil transplantation, the plants began to flower. The fruits in this study originated from flowers that were hand-pollinated within 24 h of opening. To pollinate, stamens were removed using fine point tweezers, and the anthers were rubbed directly onto the stigma of the ovule donor. Each flower was pollinated with at least 2 anthers. Pollinated flowers had their stamens intact. Crossings were recorded by pressing tagged insect pins into the cacao trunk or branch near the base of the pollinated flower’s peduncle. Successful pollination was indicated by a lack of flower senescence 48 h post-opening. Flowers from incompatible crosses senesced within 5 days post-pollination, while compatible crosses resulted in pods ripening approximately 180 days post-pollination.

### 3.5. YFP Visualization and Seed Measurements

Plant tissues were visually screened for YFP presence using a Leica M165 FC stereomicroscope equipped with a 514 nm excitation and 527 nm emission filter (JH Technologies, Fremont, CA, USA). Images were captured at 7.3–11.0× magnification using an attached Leica DFC7000 T camera and processed with Leica Application Suite X software.

To determine the inheritance pattern of transgenes, YFP expression was visualized in the seeds, radicle, or first true leaves, depending on the parents of the cross. To ensure a quick and high germination rate, the fruit pulp and seed coat were manually removed, and the seeds were placed into parafilm-sealed Petri dishes half-submerged in water. After two days, the seeds were washed and planted into 6.5 cm pots filled with wetted Sun Gro^TM^ potting mix. After fourteen days, the first leaves were fully expanded, and the tip of a leaf from each plant was manually removed for YFP expression screening.

To assess whether YFP inheritance affected seed size in the progeny of Matina 1-6 EVT1, YFP expression was viewed in each seed’s emerging radicle 2 days post-germination. Seed size was measured using ImageJ 1.52a, with length and width measured at the longest and widest points.

### 3.6. PCR Analysis

Genomic DNA was extracted from the leaf tissue of both non-transgenic and transgenic INIAPG-038 and Matina 1-6 plants using a CTAB DNA extraction protocol as described by Murray and Thompson [40]. Progeny from the cross of INIAPG-038 EVT1 with Matina 1-6 EVT1 were tested for the transgenes CaMV35Sp*-npt*II*:eyfp* and *cas9*. The presence of CaMV35Sp-nptII was identified using the primer set 35Sp 5F (5′-CAAGTGGATTGATGTGACATCTC-3′) and EYFP 1R (5′-TCGTCCTTGAAGAAGATGGTGC-3′), while *cas9* presence was tested with the primer set mCas9 4F (5′-CAGCGACGTGGACAAGCTGTTCAT-3′) and mCas9 5R (5′-AGGCGTTGAACCGATCTTCCACG-3′) (Table S1). Progeny resulting from the cross of INIAPG-038 EVT2 or EVT3 with Matina 1-6 EVT1 were tested for the transgenes NOSp*-eyfp* and *cas9*. To distinguish the presence of NOSp*-eyfp* in INIAPG-038 EVT2 or EVT3 transformed with pDDNPTYFP-2 from Matina 1-6 EVT1 transformed with pMGCC3YFP, two different forward primers, oNOSp 2F and oNOS 3F, were intentionally designed to amplify vector-specific junction regions between the sequence immediately upstream of the NOS promoter and the *eyfp* gene in each construct. The primer set oNOSp 2F (5′-TTTACGTTTGGAACTGACAGA-3′) and EYFP 1R was used for INIAPG-038 EVT2 or EVT3, while the primer set oNOSp 3F (5′-TCTAGAGGATCCCCGGGTACGA-3′) and EYFP 1R was used to identify the presence of NOSp*-eyfp* in Matina 1-6 EVT1 (Tables S1 and S2, Figure 1 and Figure 2). The same primer set was used for *cas9*, as described above. PCR reactions comprised 25 μL of DreamTaq PCR Master Mix (2x), 1 μL of the 10 μM forward primer, 1 μL of the 10 μM reverse primer, 21 μL of molecular water, and 2 μL of genomic DNA at 80 ng/μL for a total reaction volume of 50 μL.

The PCR conditions for 35Sp 5F/EYFP 1R and NOSp 2F/EYFP 1R consisted of initial denaturation at 95 °C for 2 min, followed by 10 touchdown cycles of 95 °C for 30 s, annealing for 30 s at 58 °C (−0.5 °C/cycle), extension at 72 °C for 1.5 min, followed by 20 standard cycles of 95 °C for 30 s, annealing for 30 s at 50 °C, extension at 72 °C for 40 s, final extension at 72 °C for 5 min, and 10 °C for infinity. The PCR conditions for mCas9 4F/mCas9 5R followed the same protocol except for an initial touchdown annealing temperature of 65 °C and a standard annealing temperature of 59 °C. For each PCR reaction, 10 μL was loaded onto a 1% agarose gel for electrophoresis.

### 3.7. Copy Number Analysis Using Digital Droplet PCR (ddPCR)

Transgene copy number analysis of T_0_ plants was performed using the Bio-Rad QX200 ddPCR System, as described previously, with minor modifications [41]. The primers used for the detection of target and reference genes are described in Table 1. Copies per genome of YFP and NPTII were determined by comparing target counts to those of the reference gene TcEF1a, which we confirmed to be present as a single copy (2n) in the *T. cacao* genome using BLAST (BLAST+ 2.16.0) analysis. To prepare ddPCR reaction mixtures, purified gDNA was digested with KpnI, and 10 ng of the digested template was added to each PCR reaction with 2x QX200™ ddPCR™ EvaGreen Supermix and 1 nM forward and reverse primers. Droplets were generated from a 20 uL reaction mixture and 70 uL Bio-Rad Droplet Generation Oil, following the manufacturer’s instructions. Amplification was performed with the following conditions: 10 min initial denaturation at 95 °C, 40 cycles of 1 min denaturation at 95 °C and 1 min extension at 60 °C with a 2 °C/s ramp rate, 4 °C for 5 min, and 90 °C for 10 min. Droplets were analyzed using a QX200 droplet reader and the Bio-Rad QuantaSoft software version 1.7, using the default settings for threshold determination and the quantification of positive and negative droplets. Two separate leaves were analyzed for each transgenic event, and ddPCR measurements were performed in triplicate. To compute the copy number estimations, the absolute quantification of the target was divided by that of the reference gene and multiplied by 2 to account for the two copies of EF1a in the diploid *T. cacao* genome.

### 3.8. Statistical Analysis

A χ^2^ test was used to analyze the deviation of observed segregation ratios in progeny from the expected Mendelian segregation ratios. Seed size interaction with transgene inheritance was tested using ANOVA and a Tukey–Kramer test. An α level of 0.05 was used to determine significance in all statistical analyses.

## Figures and Tables

**Table 1:** Yellow fluorescence protein (YFP) expression segregation from inter- and intra-crosses among transgenic and non-transgenic cacao plants.

	♀	INIAPG-038				Matina 1-6	
♂		NT	EVT1	EVT2	EVT3	NT	EVT1
**INIAPG-038**	**NT**	IC ^a^	IC	IC	IC	ND	15:17 ^c^
	**EVT1**	IC	IC	IC	IC	51:59 ^b^ (17:15, 13:15, 11:13, 10:16)	ND
	**EVT2**	IC	IC	IC	IC	14:11 ^c^	ND
	**EVT3**	IC	IC	IC	IC	15:11 ^c^	ND
**Matina 1-6**	**NT**	0:177 ^a^ (0:42, 0:38, 0:37, 0:29, 0:31)	63:53 ^b^ (17:15, 26:17, 10:16, 10:5)	11:14 ^c^	15:12 ^c^	0:64 ^a^ (0:25, 0:11, 0:28)	34:29 ^c^ (13:9, 14:14, 7:6)
	**EVT1**	25:27 ^b^ (15:13, 10:14)	55:36:40 ^d^ (15:8:8 *, 12:5:6, 15:11:12, 13:12:14)	14:4 ^e,^*	6:2 ^e^	26:27 ^c^ (13:7, 7:14, 6:6)	119:42 ^e^ (11:4, 20:11, 8:2, 18:7, 21:5, 18:4 *, 23:9)

NT: non-transgenic, IC: incompatible, ND: not determined. ^a–e^ Expected segregation ratios for YFP expression: ^a^ all negative, ^b^ 1:1 (strong: no expression), ^c^ 1:1 (weak: no expression), ^d^ 2:1:1 (strong, weak: no expression), ^e^ 3:1 (weak: no expression). Numbers in parentheses indicate the YFP expression segregation ratios observed in radicles from individual germinating T_1_ seeds. Relative YFP fluorescence intensity values derived from these assessments were used to define expression categories as follows: strong (>80th percentile), moderately strong (30th–80th percentile), and low (<30th percentile). No moderately strong YFP expression was observed in T_1_ progeny plants. * Detailed segregation data for YFP expression and PCR analyses are provided in Table S2. All experimental segregation ratios were not significantly different than the expected segregation ratios using an χ^2^ test at *p* ≤ 0.05.

**Table 2:** Germination rates and size of seeds from individual cacao pods produced from non-transgenic plants and crosses between transgenic and non-transgenic plants.

Ovule Donor	Pollen Donor	Germination Rate	Average Seed Length (cm) *	Average Seed Width (cm) **
INIAPG-038	Matina 1-6	31/31	1.78	0.83
INIAPG-038	Matina 1-6	29/31	2.08	1.03
INIAPG-038	Matina 1-6	37/38	2.06	1.13
INIAPG-038	Matina 1-6 EVT 1	24/24	2.24	1.15
INIAPG-038 EVT1	Matina 1-6 EVT 1	38/38	2.36	1.16
INIAPG-038 EVT1	Matina 1-6 EVT 1	39/39	2.10	1.10
INIAPG-038 EVT1	Matina 1-6 EVT 1	27/27	2.20	1.07
INIAPG-038 EVT2	Matina 1-6 EVT 1	22/23	2.18	1.07
INIAPG-038 EVT3	Matina 1-6 EVT 1	8/12	2.41	1.15
Matina 1-6	Matina 1-6	11/12	2.18	1.13
Matina 1-6	INIAPG-038 EVT1	25/26	2.13	1.05
Matina 1-6	Matina 1-6 EVT 1	21/22	1.89	1.06
Matina 1-6	Matina 1-6 EVT 1	20/20	1.99	1.06
Matina 1-6	Matina 1-6 EVT 1	12/12	2.07	1.02
Matina 1-6 EVT 1	Matina 1-6 EVT 1	31/32	1.89	0.91
	Average	375/387 (97%)	2.10	1.06

* and ** indicate no statistical difference (ANOVA, *p* > 0.05) by column; PSU used a single event.

**Table 3:** Distribution of seed sizes within single pods of cacao by transgene inheritance based on expression of yellow fluorescence protein (YFP).

Ovule Donor	Pollen Donor	Inheritance Ratio	YFP from INIAPG-038 EVT1 with or Without Matina 1-6 EVT1 ***		YFP from Only Matina 1-6 EVT1 (Heterozygous) ***		No YFP from Either Parent (Null Segregant) ***	
		YFP Expression Segregation Ratio *	Length (cm) **	Width (cm) **	Length (cm)	Width (cm)	Length (cm)	Width (cm)
INIAPG-038 EVT1	Matina 1-6 EVT1	15:11:12	2.42	1.19	2.41	1.19	2.26	1.09
INIAPG-038 EVT1	Matina 1-6 EVT1	11:8:8	2.10	1.00	2.36	1.19	2.19	1.04
Matina 1-6	Matina 1-6 EVT1	0:7:14	N/A	N/A	2.00	1.15 a	1.84	1.04 b
Matina 1-6	Matina 1-6 EVT1	0:13:7	N/A	N/A	2.06	1.14 a	1.86	0.96 b

* Number of seeds in a pod with high YFP expression, moderate YFP expression, or no YFP expression. ** Means followed by the same or no letter indicate no significant difference (ANOVA, *p* > 0.05) compared to corresponding measurements in other columns of a row. *** Visual YFP expression was evaluated in the radicles of individual germinating T_1_ progeny seeds.

## Data Availability

Data are contained within the article.

## Supplementary Materials

The following supporting information can be downloaded at: https://www.mdpi.com/article/10.3390/plants15040642/s1, Table S1: Primer sets used for selecting transgenic plants and detecting YFP expression in four different transgenic cacao events and their progeny. Table S2: Analysis of YFP expression and transgene inheritance in T_1_ progeny plants resulting from self-pollinated Matina 1-6 EVT1 and crosses between INIAPG-038 EVT1 or EVT2 and Matina 1-6 EVT1. Table S3. Primer sets used for copy number analysis by digital droplet PCR (ddPCR). Figure S1: YFP expression in T_0_ *Theobroma cacao* L. plants. Figure S2. Western blot analysis of YFP protein levels in leaves of transgenic INIAPG-038 and Matina 1-6 cacao plants [42,43].
