# Supplementary material for: Stable Inheritance of Transgene and Yellow Fluorescent Protein Gene Expression in Progeny of Transgenic Cacao (Theobroma cacao) Plants
Source: Plants (Basel). 2026 Feb 18;15(4):642. doi: 10.3390/plants15040642 (PMC12944052; doi:10.3390/plants15040642)
Supplement: Supplementary file 1 [file plants-15-00642-s001.zip › plants-4077406-supplementary.pdf]

plants

Supplementary Information

Table S1. Primer sets used for selecting transgenic plants and detecting YFP expression in four different transgenic cacao events and their progeny.

Name	Sequence (5′ to 3′)	Band Size (bp)
35Sp 5F	CAAGTGGATTGATGTGACATCTC	1281
EYFP 1R	TCGTCCTTGAAGAAGATGGTGC	
oNOSp 2F	TTTACGTTTGGAACTGACAGA	593
EYFP 1R	TCGTCCTTGAAGAAGATGGTGC	
oNOSp 3F	TCTAGAGGATCCCCGGGTACGA	594
EYFP 1R	TCGTCCTTGAAGAAGATGGTGC	
mCas9 4F	CAGCGACGTGGACAAGCTGTTCAT	1235
mCas9 5R	AGGCGTTGAACCGATCTTCCACG	

Table S2. Analysis of YFP expression and transgene inheritance in T1 progeny plants resulting from self-pollinated Matina 1-6 EVT1 and crosses between INIAPG-038 EVT1 or EVT2 and Matina 1-6 EVT1. Reciprocal crosses between INIAPG-038 EVT1 and Matina 1-6 EVT1 yielded a 2:1:1 segregation (strong: weak: no expression), consistent with the expected 3:1 positive-to-negative ratio. The strong CaMV 35S–driven YFP in INIAPG-038 EVT1 masked the weaker NOSp::TMV Ω–driven signal, making progeny inheriting both transgenes indistinguishable from those carrying only the CaMV 35S cassette. No moderately strong YFP expression was observed in T1 progeny plants. Visual YFP expression was assessed using identical settings under a fluorescence microscope.

Ovule Donor	Pollen Donor	Visual YFP Expression	PCR Amplicon (35Sp 5F/EYFP 1R)	PCR Amplicon (oNOSp 2F/EYFP 1R)	PCR Amplicon (oNOSp 3F/EYFP 1R)	PCR Amplicon (mCas9 4F/mCas9 5R)		
INIAPG-038 EVT1	Matina 1-6 EVT1	+	-	ND	+	+		
INIAPG-038 EVT1	Matina 1-6 EVT1	+	-	ND	+	+		
INIAPG-038 EVT1	Matina 1-6 EVT1	+	-	ND	+	+		
INIAPG-038 EVT1	Matina 1-6 EVT1	+	-	ND	+	+		
INIAPG-038 EVT1	Matina 1-6 EVT1	+	-	ND	+	+		
INIAPG-038 EVT1	Matina 1-6 EVT1	+	-	ND	+	+		
INIAPG-038 EVT1	Matina 1-6 EVT1	+	-	ND	+	+		
INIAPG-038 EVT1	Matina 1-6 EVT1	+	-	ND	+	+		
INIAPG-038 EVT1	Matina 1-6 EVT1	+++++	+	ND	-	-		
INIAPG-038 EVT1	Matina 1-6 EVT1	-	-	ND	-	-		
INIAPG-038 EVT1	Matina 1-6 EVT1	-	-	ND	-	-		
INIAPG-038 EVT1	Matina 1-6 EVT1	+++++	+	ND	+	+		
INIAPG-038 EVT1	Matina 1-6 EVT1	-	-	ND	-	-		
INIAPG-038 EVT1	Matina 1-6 EVT1	-	-	ND	-	-		
INIAPG-038 EVT1	Matina 1-6 EVT1	-	-	ND	-	-		
INIAPG-038 EVT1	Matina 1-6 EVT1	-	-	ND	-	-		
INIAPG-038 EVT1	Matina 1-6 EVT1	-	-	ND	-	-		
INIAPG-038 EVT1	Matina 1-6 EVT1	-	-	ND	-	-		
INIAPG-038 EVT1	Matina 1-6 EVT1	+++++	+ (weak)	ND	+	+		
INIAPG-038 EVT1	Matina 1-6 EVT1	+++++	+ (faint)	ND	-	-		
INIAPG-038 EVT1	Matina 1-6 EVT1	+++++	+	ND	+	+		
INIAPG-038 EVT1	Matina 1-6 EVT1	+++++	+ (weak)	ND	+	+		
INIAPG-038 EVT1	Matina 1-6 EVT1	+++++	+	ND	+	+		
INIAPG-038 EVT1	Matina 1-6 EVT1	+++++	+	ND	-	-		
INIAPG-038 EVT1	Matina 1-6 EVT1	+++++	+	ND	-	-		
INIAPG-038 EVT1	Matina 1-6 EVT1	+++++	+ (faint)	ND	-	-		
INIAPG-038 EVT1	Matina 1-6 EVT1	+++++	+	ND	-	-		
INIAPG-038 EVT1	Matina 1-6 EVT1	+++++	+	ND	-	-		
INIAPG-038 EVT1	Matina 1-6 EVT1	+++++	+	ND	-	-		
INIAPG-038 EVT1	Matina 1-6 EVT1	+++++	+	ND	-	-		
INIAPG-038 EVT1	Matina 1-6 EVT1	+++++	+	ND	-	-	15:8:8	strong: weak: no expression
INIAPG-038 EVT2	Matina 1-6 EVT1	+	ND	-	+	ND		
INIAPG-038 EVT2	Matina 1-6 EVT1	-	ND	-	-	-		
INIAPG-038 EVT2	Matina 1-6 EVT1	+	ND	+	+	+		
INIAPG-038 EVT2	Matina 1-6 EVT1	+	ND	+	+	+		
INIAPG-038 EVT2	Matina 1-6 EVT1	+	ND	+	-	-		
INIAPG-038 EVT2	Matina 1-6 EVT1	+	ND	+	+	+		
INIAPG-038 EVT2	Matina 1-6 EVT1	+	ND	-	+	+		
INIAPG-038 EVT2	Matina 1-6 EVT1	+	ND	+	+	+		
INIAPG-038 EVT2	Matina 1-6 EVT1	+	ND	+	-	-		
INIAPG-038 EVT2	Matina 1-6 EVT1	-	ND	-	-	-		
INIAPG-038 EVT2	Matina 1-6 EVT1	-	ND	-	-	-		
INIAPG-038 EVT2	Matina 1-6 EVT1	+	ND	-	+	+		
INIAPG-038 EVT2	Matina 1-6 EVT1	+	ND	-	+	+		
INIAPG-038 EVT2	Matina 1-6 EVT1	+	ND	+	-	-		
INIAPG-038 EVT2	Matina 1-6 EVT1	+	ND	-	+	+		
INIAPG-038 EVT2	Matina 1-6 EVT1	-	ND	-	-	-		
INIAPG-038 EVT2	Matina 1-6 EVT1	+	ND	+	-	-		
INIAPG-038 EVT2	Matina 1-6 EVT1	+	ND	+	+	+	14:4	weak: no expression
			ND					
Matina 1-6 EVT1	Matina 1-6 EVT1	+	ND	ND	+	+		
Matina 1-6 EVT1	Matina 1-6 EVT1	+	ND	ND	+	+		
Matina 1-6 EVT1	Matina 1-6 EVT1	+	ND	ND	+	+		
Matina 1-6 EVT1	Matina 1-6 EVT1	+	ND	ND	+	+		
Matina 1-6 EVT1	Matina 1-6 EVT1	+	ND	ND	+	+		
Matina 1-6 EVT1	Matina 1-6 EVT1	+	ND	ND	+	+		
Matina 1-6 EVT1	Matina 1-6 EVT1	+	ND	ND	+	+		
Matina 1-6 EVT1	Matina 1-6 EVT1	+	ND	ND	+	+		
Matina 1-6 EVT1	Matina 1-6 EVT1	+	ND	ND	+	+		
Matina 1-6 EVT1	Matina 1-6 EVT1	+	ND	ND	+	+		
Matina 1-6 EVT1	Matina 1-6 EVT1	+	ND	ND	+	+		
Matina 1-6 EVT1	Matina 1-6 EVT1	+	ND	ND	+	+		
Matina 1-6 EVT1	Matina 1-6 EVT1	+	ND	ND	+	+		
Matina 1-6 EVT1	Matina 1-6 EVT1	-	ND	ND	-	-		
Matina 1-6 EVT1	Matina 1-6 EVT1	+	ND	ND	+	+		
Matina 1-6 EVT1	Matina 1-6 EVT1	+	ND	ND	+	+		
Matina 1-6 EVT1	Matina 1-6 EVT1	+	ND	ND	+	+		
Matina 1-6 EVT1	Matina 1-6 EVT1	+	ND	ND	+	+		
Matina 1-6 EVT1	Matina 1-6 EVT1	-	ND	ND	-	-		
Matina 1-6 EVT1	Matina 1-6 EVT1	-	ND	ND	-	-		
Matina 1-6 EVT1	Matina 1-6 EVT1	-	ND	ND	-	-		
Matina 1-6 EVT1	Matina 1-6 EVT1	+	ND	ND	+	+	18:4	weak: no expression

Table S3. Primer sets used for copy number analysis by digital droplet PCR (ddPCR).

Name	Sequence (5′ to 3′)
EF1a-ddPCR-F	TTGATGGCATATGCAAAGATTCTGG
EF1a-ddPCR-R	CAAGTGGGTGGAAGTTATTGCCC
NPTII-ddPCR-F	CTGCTTGCCGAATATCATGGTGG
NPTII-ddPCR-R	TCTTCAGCAATATCACGGGTAGCC
YFP-ddPCR-F	CGAAGGCTACGTCCAGGAGC
YFP-ddPCR-R	GTCGATGCCCTTCAGCTCG

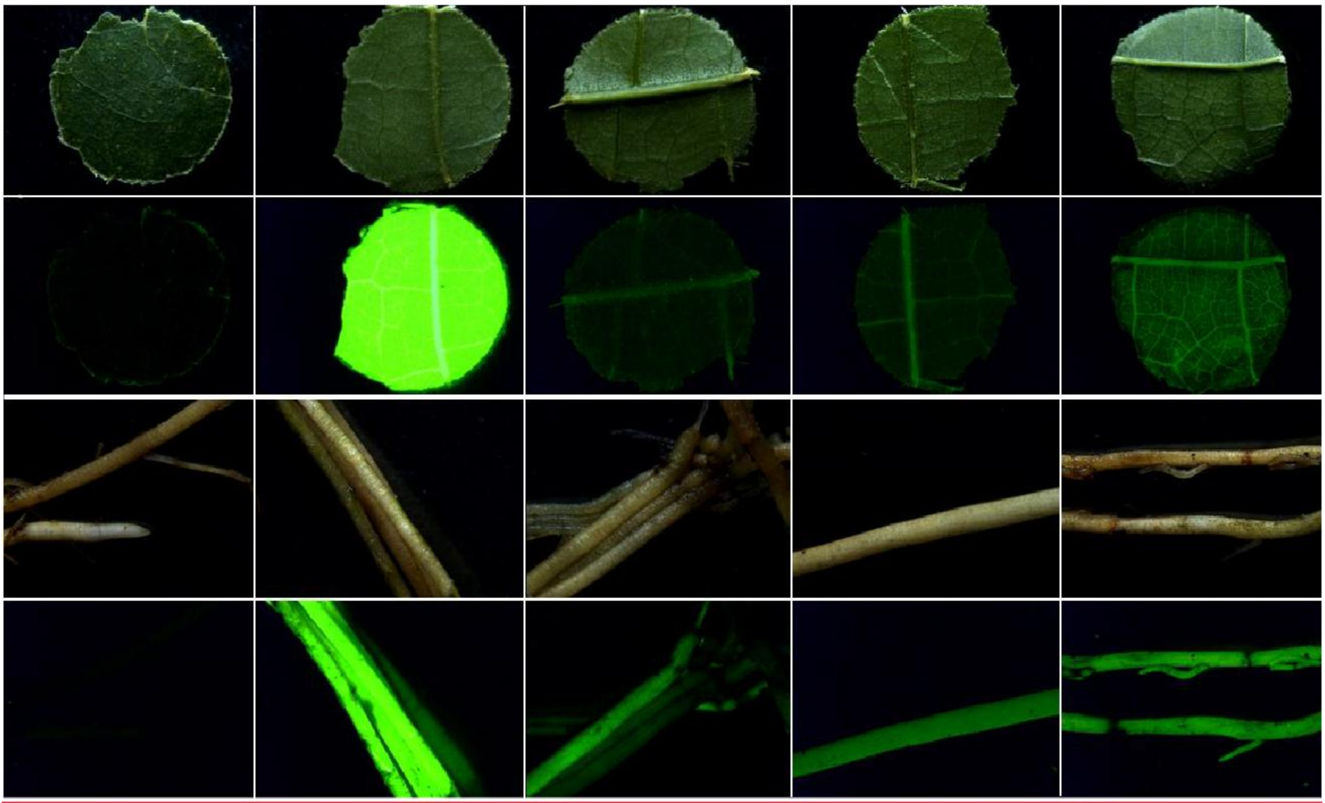

Figure S1. YFP expression in T_0 *Theobroma cacao* L. plants. (A) Top row and third row: YFP filter; second row and bottom row: brightfield. From left to right: leaf and root samples from non-transgenic INIAPG-038, INIAPG-038 EVT 1, INIAPG-038 EVT 2, INIAPG-038 EVT 3, and Matina 1-6 EVT1.

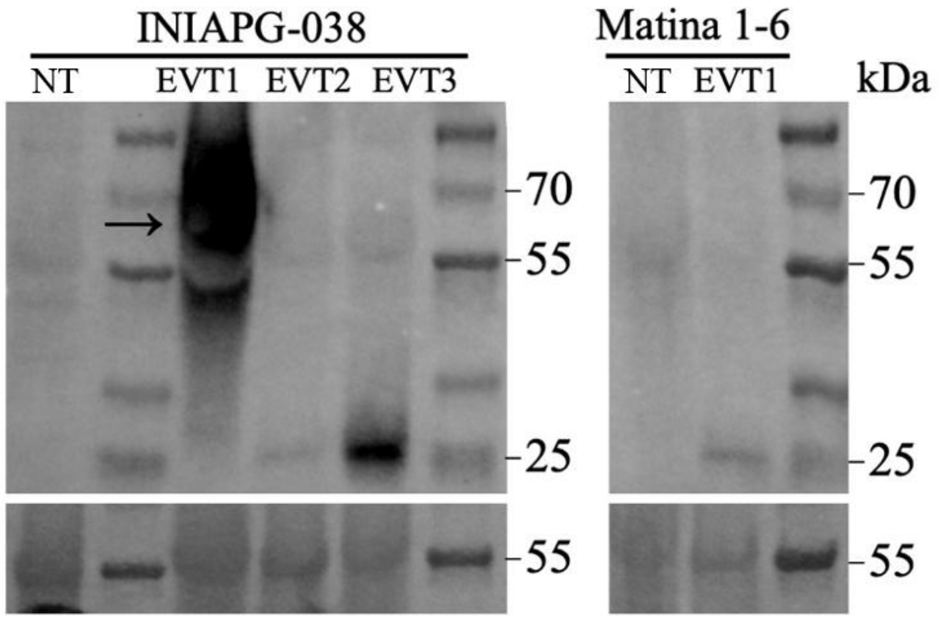

Figure S2. Western blot analysis of YFP protein levels in leaves of transgenic INIAPG-038 and Matina 1-6 cacao plants. The upper panel shows immunodetection of YFP in young leaf samples from six cacao plants using an anti-YFP antibody. The lower panel shows the corresponding Rubisco large subunit signal as a loading control. Samples include one non-transgenic INIAPG-038 plant (NT), three independent transgenic INIAPG-038 events (EVT1, EVT2 and EVT3), one non-transgenic Matina 1-6 plant (NT), and one transgenic Matina 1-6 plant (EVT1). YFP expression was detected only in the transgenic lines.

Western Blot Analysis of YFP Expression Levels

Immunoblot assays were performed as previously described (Qi et al., 2018), with minor modifications. Briefly, total protein was extracted from cacao leaves using 2× Laemmli buffer containing 5% β-mercaptoethanol. Proteins were separated on a 4–15% precast polyacrylamide gel (Bio-Rad, Hercules, CA), transferred to nitrocellulose membranes, and then probed with a mouse anti-YFP antibody (1:1,000; Biosensis Pty Ltd., Thebarton, SA, Australia). Antigens were detected using the SuperSignal™ West Pico PLUS Chemiluminescent Substrate (Thermo Fisher Scientific, Waltham, MA) and imaged with a ChemiDoc XRS+ system (Bio-Rad, Hercules, CA). Ponceau S staining was used as a loading control. Relative YFP expression was quantified from western blot images using ImageJ software (Schneider et al., 2012).

References

Qi, T., Seong, K., Thomazella, D.P.T., Kim, J.R., Pham, J., Seo, E., Cho, M.-J., Schultink, A., Staskawicz, B.J. NRG1 functions downstream of EDS1 to regulate TIR-NLR-mediated plant immunity in *Nicotiana benthamiana, Proc. Natl. Acad. Sci.* **2018,** *115, E10979-E10987.*

Schneider, C.A., Rasband, W.S., Eliceiri, K.W. NIH Image to ImageJ: 25 years of image analysis. *Nat. Methods*, ***2012,*** 9, 671–675.
